# Bilateral cavernous sinus dural arteriovenous fistula with initial ocular symptom: A case report

**DOI:** 10.1097/MD.0000000000027892

**Published:** 2021-11-19

**Authors:** Qian Zhang, Xiao-Ling Xu, Ya-Li Sun, Zi-Wei Wang, Xian-Liang Lai, Yu Xiong

**Affiliations:** Department of Ophthalmology, the Second Affiliated Hospital of Nanchang University, Nanchang, Jiangxi Province, China.

**Keywords:** cavernous sinus dural arteriovenous fistula, digital subtraction angiography, intravascular embolization, treatment

## Abstract

**Rationale:**

Cavernous sinus dural arteriovenous fistula (CSDAVF) is a rare intracranial vascular malformation. Because of its complicated clinical manifestations, it is easy to miss or misdiagnose CSDAVF.

**Patient concerns:**

A 42-year-old female had chief complaint that the right eyeball had conjunctival congestion for half a year. She was given levofloxacin eye drops to treat the right eye with anti-inflammatory treatment, but the symptoms did not improve. Cranial magnetic resonance and cerebrovascular imaging showed that the right lateral rectus muscle was slightly enlarged, the right eyeball was prominent, but there was no abnormality in the brain.

**Diagnoses:**

Based on clinical and imaging examinations and digital subtraction angiography (DSA), she was diagnosed as low-flow CSDAVF.

**Interventions:**

The patient received interventional embolization with transvenous combined arterial approach using coils and Onyx liquid glue.

**Outcomes:**

The patient's exophthalmos and congestion symptoms were improved.

**Conclusion:**

DAS is the gold standard for the diagnose of CSDAVF. Intravascular embolization interventional therapy is an effective treatment for CSDAVF.

## 1 Introduction

Dural arteriovenous fistula indicates a lesion with an arteriovenous shunt in the dural membrane, and if it occurs in the cavernous sinus (CS), it is called cavernous sinus dural arteriovenous fistula (CSDAVF).^[1]^ CSDAVF is a rare intracranial vascular malformation, and is usually characterized by abnormal shunts between the arterial and venous circulation on the dura mater within or close to the walls of a dural sinus. These fistulas arise spontaneously and represent about 10% to 15% of all intracranial arteriovenous shunts.^[2]^

Patients with CSDAVF usually present with the signs related to the eyes, including chemosis, exophthalmos, glaucoma, and palpebral swelling, but the symptoms of CSDAVF depend on the location of the shunt and the type of venous drainage.^[3]^ Currently, endovascular technique is a common treatment method for patients with symptomatic CSDAVF. Both transvenous and transarterial approaches of endovascular embolization are accepted as effective and safe treatment for CSDAVF.^[4]^

In this case report, we retrospectively analyzed a case of CSDAVF with ocular symptoms as initial symptoms, focusing on the diagnosis, clinical course and treatment outcome.

## 2 Case presentation

A 42-year-old female had chief complaint that the right eyeball had conjunctival congestion for half a year. Six months ago, the patient found that her right eye had redness, prominent eyeballs and decreased vision, and had occasional headaches. She went to the local hospital and was diagnosed with conjunctivitis, and thyroid hormone test was normal, and she had no history of hypertension, diabetes, hyperlipidemia, hepatitis, tuberculosis, and drug allergy. She was given levofloxacin eye drops to treat the right eye with anti-inflammatory treatment, but the symptoms did not improve. Therefore, she went to our hospital for further treatment. Cranial magnetic resonance and cerebrovascular imaging showed that the right lateral rectus muscle was slightly enlarged, the right eyeball was prominent, but there was no abnormality in the brain.

**Figure 1:**
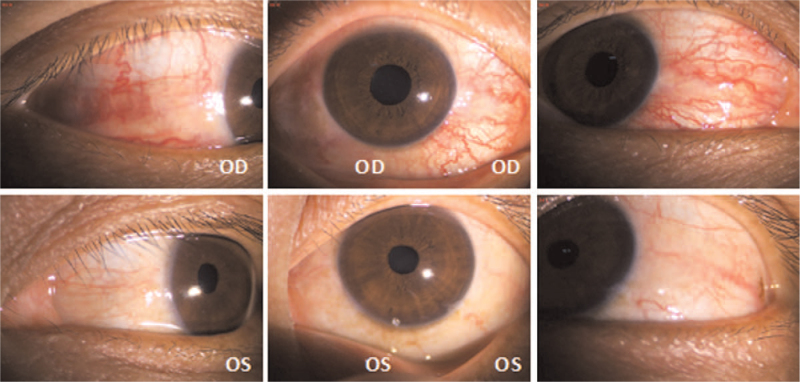
Anterior segment photography showed spiral-shaped bulbar conjunctival blood vessels and enlarged right eye pupil.

**Figure 2:**
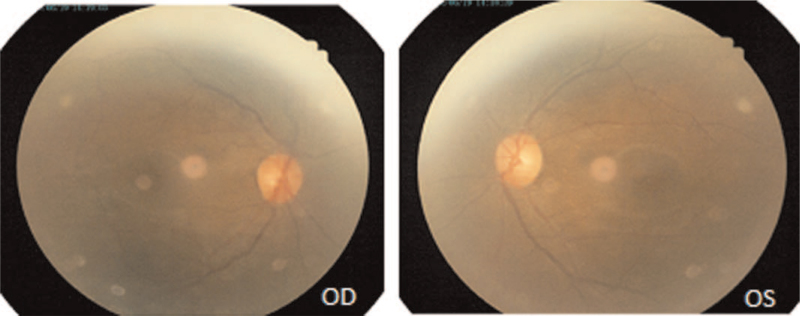
Fundus photography showed the expansive retinal vein.

**Figure 3:**
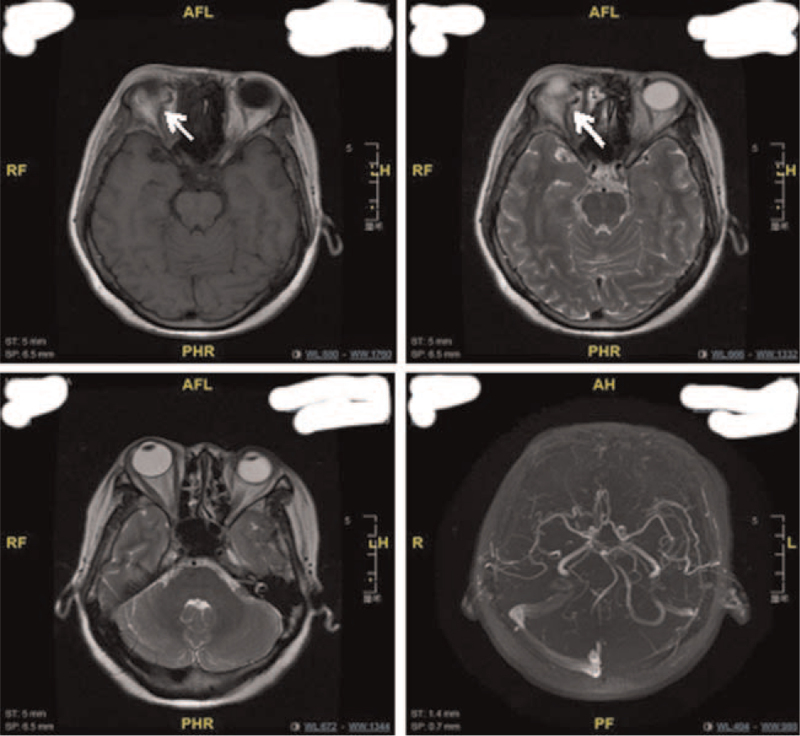
Magnetic resonance showed enlarged superior ocular vein (white arrows) on the right eye.

**Figure 4:**
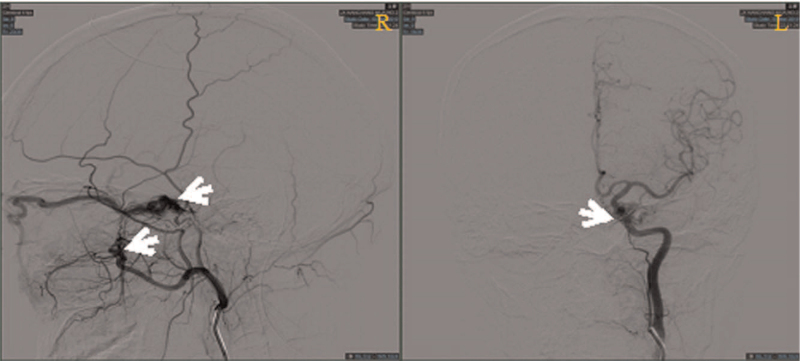
DSA showed that the right middle cerebral artery was stenosis. The smaller distal blood vessels were replaced by messy small “moyamoya disease-like” arteries (white arrows). The superior ocular veins in the cavernous sinus area on both sides were blocked and dilated, especially on the right side.

Physical examination: T 36.5°C P 68 times/min R 19 times/min BP 127/67 mm Hg. Consciousness was clear, GCS scored 15 points (E4V5M6). Specialist examination: visual acuity: OD: 0.1, −2.25DS = −2.00DC × 170°–>0.4; OS: 0.1, −1.75DS = −1.25DC × 175° –> 0.8; The conjunctiva of both eyes had hyperemia, and the blood vessels were spirally tortuous and dilated, especially in the right eye. The pupil diameter of right eye was about 4 mm, while that of the left eye was about 3 mm, and both eyes were sensitive to the light reflection (Fig. 1). Retinal vein of both eyes were slightly tortuous, the arteriovenous ratio was 1:2 (Fig. 2). Intraocular pressure: OD: 14.0 mm Hg, OS: 13.0 mm Hg. The degree of eyeball protrusion: OD: 18.0 mm, OS: 14.0 mm, orbital distance: 103 mm. There was no pulsating vascular murmur. Head down test was negative. Magnetic resonance showed enlarged veins on the right side of the eye (Fig. 3). Thyroid hormone test results were as follows: triiodothyronine (T3) 1.01 ng/mL, thyroid hormone 6.8 ug/dL, hypersensitivity thyrotropin 1.482 mIU/L. Digital subtraction angiography (DSA) showed that the right middle cerebral artery was stenosis. The distal blood vessels were replaced by messy and small “moyamoya disease-like” arteries; the superior ocular veins in the cavernous sinus area on both sides were obstructed and dilated, especially on the right side, and the blood supply artery was a branch of the right maxillary artery blood vessels; the left internal carotid artery and the left vertebral artery were normal, with clear visualization and no clear arteriovenous shunt (Fig. 4). Based on these results, the patient was diagnosed as CSDAVF.

**Figure 5:**
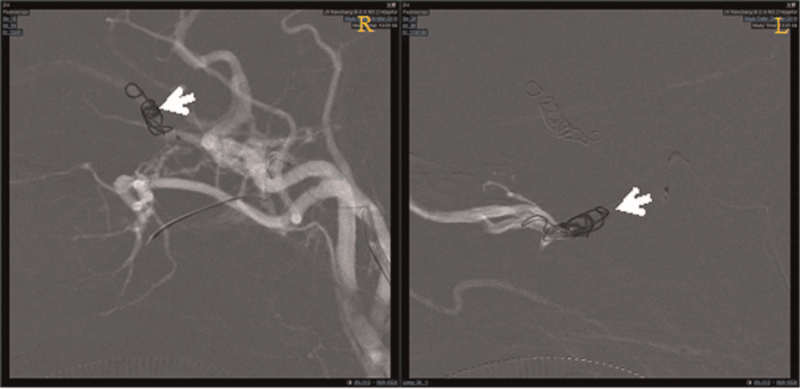
Intravascular embolization therapy showed that the joint of the ocular vein and cavernous sinus can be embolized by releasing the coil through the microcatheter.

**Figure 6:**
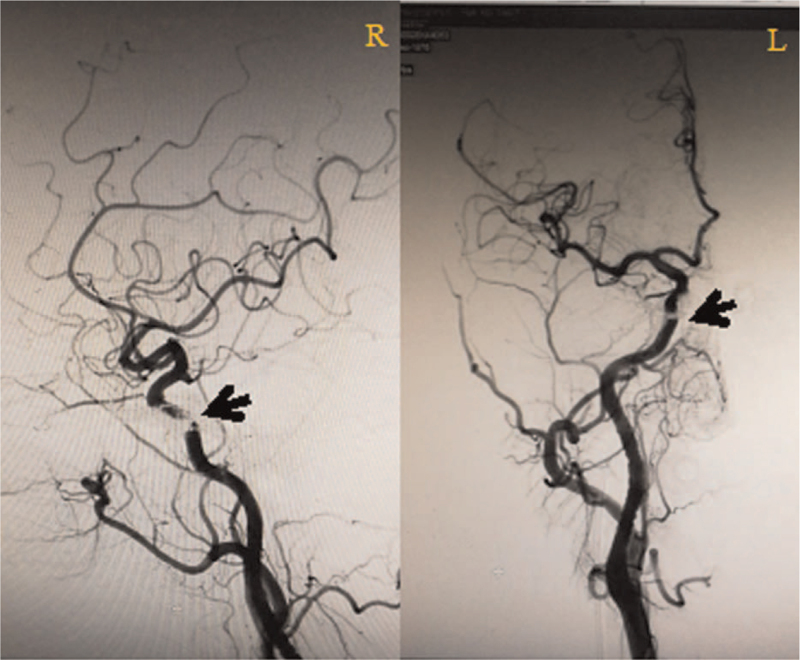
After embolization, DSA showed that the injected glue (black arrow) was completely dispersed in the sinus, and the arteriovenous fistula was completely occluded, indicating that CSDAVF was successfully blocked.

Next, transvenous combined arterial approach was performed using coils and Onyx liquid glue for interventional embolization (Figs. 5 and 6). The patient's exophthalmos and congestion symptoms were improved on the first day after surgery, and her vision did not change. At the time of discharge, the patient was conscious, self-reported diplopia, GCS 15 points, right exophthalmos and hyperemia disappeared, bilateral pupils were 3.0 mm, sensitive to light, limbs activity was normal, muscle strength level 5, muscle tension was normal, the physiological reflex was present, and the meningeal irritation and pathological signs were not elicited.

Diplopia after surgery may be related to local tissue edema compressing the oculomotor nerve, which relieved 3 weeks after surgery, and her vison acuity of right eye recovered to 0.5. After half a year of follow-up, mild headache still persisted, which may be caused by the compression of the embolic material on the first branch of the trigeminal nerve. The patient used carbamazepine for pain relief.

## 3 Discussion

CSDAVF is an abnormal communication that occurs between the cavernous sinus and the external carotid artery and/or the dural branch of the internal carotid artery. CSDAVF is a rare clinical cranial cerebrovascular malformations.^[5]^ CS is the most frequent area in DAVF of Asian, accounting for about 48%, and the transverse sinus is the most common site of DAVF in the Western countries.^[6]^ The main drainage vein of CSDAVF is the superior ocular vein, so ocular manifestations are the most common symptoms. Arterial blood flows into the cavernous sinus through the fistula and flows into the orbit through the superior ocular vein to the front, which can cause increased venous pressure, venous blood reflux, and ocular ischemia. Eyelid and bulbar conjunctival hyperemia and edema, and dilation of the central retinal vein and papillary edema can be seen in the fundus. The resistance to the outflow of aqueous humor increases, and the scleral venous pressure also increases, which can increase intraocular pressure. Papilledema and increased intraocular pressure will cause vision loss. Arterial blood flows into the cavernous sinus, causing the cavernous sinus to expand and damage the oculomotor nerve, trochlear nerve, trigeminal nerve, and abductor nerve passing through the cavernous sinus, resulting in symptoms and signs such as diplopia, extraocular muscle palsy, and pain.^[7]^ Due to the special anatomical structure and drainage characteristics of cavernous sinus venous drainage, clinical manifestations of CSDAVF are complicated, and patients with bilateral CSDAVF are even rare. Most patients are first diagnosed in ophthalmology due to ocular symptoms. Due to the lack of knowledge about CSDAVF, it is easy to miss and misdiagnose CSDAVF.

The clinical manifestations of CSDAVF are also related to the flow of arteriovenous fistula. High-flow CSDAVF often has typical ocular signs, pulsatile tinnitus and blood flow murmurs. Low-flow CSDAVF is also known as “Red Eye Short-circuit Syndrome,” and mostly occurs in middle-aged and elderly women without a history of trauma. It mainly manifests as dilated and tortuous superior scleral and conjunctival veins, elevated superior scleral venous pressure and intraocular pressure.^[8]^ In this case report, the patient's conjunctival and fundus veins were dilated and tortuous, without pulsatile murmurs and tinnitus, and she was diagnosed with low-flow CSDAVF.

In addition to the symptoms and signs, the diagnosis of CSDAVF depends on imaging examinations.^[9]^ CT or MRI shows dilated superior ocular veins, but CT and MRI examinations are usually very atypical, and it is easy to miss or delay the diagnosis. Currently, DSA is gold standard for the diagnosis of CSDAVF.^[10]^

CSDAVF should be differentiated from the following diseases:^[11]^

1. Inflammatory pseudotumor: CT shows irregularly shaped and high-density space-occupying lesions behind the eyeball. Sometimes the upper ocular veins are dilated, but it is relatively mild. There is no enlargement of CS in the inflammatory pseudotumor.
2. Thyroid-related ophthalmopathy: there is often abnormal thyroid function or abnormal regulation. The imaging usually shows spindle-shaped swelling of multiple extraocular muscles in the eyes, and there is no obvious change in tendons and muscle. There is also no abnormal enlargement of CS in thyroid-related ophthalmopathy.
3. Internal carotid cavernous sinus fistula: Patients may have headache, pulsating exophthalmos, murmurs, bulbar conjunctival edema, congestion, eye movement disorders, visual disturbances, nervous system dysfunction, intracranial hemorrhage and epistaxis. It is difficult to distinguish it from CDAVF and requires angiography to confirm the diagnosis.
4. Cavernous sinus thrombosis: Although patients may have bulbar conjunctival congestion and edema, exophthalmos, and eye movement disorders, there will be no murmurs. During the course of the disease, there is often a history of facial furuncle carbuncle or systemic infection, which can be further identified by angiography.
5. Chronic conjunctivitis: This disease may present with conjunctival hyperemia and edema, but there is no intracranial murmur, exophthalmos, and eye movement disorder can be distinguished.

The purpose of treatment for CSDAVF is to restore normal intracranial blood circulation. Current treatment methods are diverse, mainly including carotid artery compression, Gamma Knife surgery, endovascular embolization, surgery and stereotactic radiotherapy.^[12,13]^ Carotid artery compression therapy is only effective for CSDAVF with small fistulas, low blood flow, and blood supplied by the external carotid artery, but this method does not have a good effect on both the internal and external carotid arteries.^[14]^ At present, endovascular embolization is the most effective method for the treatment of CSDAVF.^[15–17]^ Because CS is separated by many fiber trabeculae, it is difficult to achieve dense embolization in the sinus using spring coils, and coil plus glue is the first choice for the treatment of CSDAVF.^[18,19]^ The coil filled in the sinus acts as a support, which can reduce the blood flow velocity in the sinus and the fistula, thereby preventing the glue from entering the important drainage veins such as the scoliosis vein and the superior ocular vein. The Onyx glue is a non-sticky glue that can be injected slowly in a relatively sufficient time, and it can be fully dispersed into the various multilocular structures in order to block the sinus.^[20]^ In this case, CSDAVF was embolized by the venous system, and coils and Onyx glue were selected for embolization. Postoperative imaging showed that the fistula was completely occluded, but long-term efficacy remains to be investigated.

Yang et al, compared prognostic factors and treatment effectiveness of endovascular treatment (EVT) and stereotaxic gamma-knife radiosurgery (GKRS) for different CSDAVF types. The results showed that EVT was the first choice of treatment, especially for restrictive CSDAVFs, but GKRS had lower complication rate with similar therapeutic effects for proliferative type fistulas.^[21]^ Churojana et al, analyzed 165 consecutive patients diagnosed with CSDAVF and bilateral CSDAVF was detected in 43 patients (26%). The most common complication of coil embolization in the treatment of CSDAVF is cranial nerve palsy, including new cranial nerve palsy and exacerbation of the original cranial nerve palsy.^[16]^ Rhim et al reported that of 17 cases of bilateral CSDAVF, 7 cases (41.2%) had increased cranial nerve palsy after embolization.^[15]^ Therefore, excessive use of spring coils and Onyx glue should be avoided during the operation.

## 4 Conclusion

If imaging findings showed the involvement or thickening of eye veins, thickening of extraocular muscles, and enlarged cavernous sinus, we should consider the possibility of CSDAVF. DSA is the gold standard for the diagnosis of CSDAVF. Intravascular embolization therapy is an effective treatment for CSDAVF.

## Author contributions

**Conceptualization:** Yu Xiong.

**Investigation:** Qian Zhang, Xiao-Ling Xu, Ya-Li Sun, Zi-Wei Wang, Xian-Liang Lai.

**Writing – original draft:** Yu Xiong.

**Writing – review & editing:** Yu Xiong.
